# Primary Biliary Cholangitis (PBC)-Autoimmune Hepatitis (AIH) Variant Syndrome: Clinical Features, Response to Therapy and Long-Term Outcome

**DOI:** 10.3390/jcm12227047

**Published:** 2023-11-11

**Authors:** Markus Graf, Christian M. Lange, Mona M. Langer, Jörn M. Schattenberg, Jessica Seessle, Julia Dietz, Annika Vermehren, Florian A. Michael, Antonia Mondorf, Stefan Zeuzem, Anita Pathil, Christiana Graf

**Affiliations:** 1 Department of Internal Medicine I, University Hospital Frankfurt, 60596 Frankfurt am Main, Germany; markus.m.graf@tum.de (M.G.); julia.dietz@kgu.de (J.D.); annika.vermehren@kgu.de (A.V.); florian-alexander.michael@kgu.de (F.A.M.); antonia.mondorf@kgu.de (A.M.); stefan.zeuzem@kgu.de (S.Z.); anita.pathil-warth@kgu.de (A.P.); 2 Department of Internal Medicine II, University Hospital Munich, 81377 Munich, Germany; christian.lange@med.uni-muenchen.de (C.M.L.); mona.langer@med.uni-muenchen.de (M.M.L.); 3 Department of Internal Medicine I, University Medical Center Mainz, 55131 Mainz, Germany; joern.schattenberg@unimedizin-mainz.de; 4 Department of Internal Medicine IV, University of Heidelberg, 69120 Heidelberg, Germany; jessica.seessle@med.uni-heidelberg.de

**Keywords:** autoimmune hepatitis (AIH), primary biliary cholangitis (PBC), variant syndrome (VS), Paris criteria, immunosuppression, liver cirrhosis, portal hypertension

## Abstract

Introduction: Standardization of diagnostic criteria of autoimmune hepatitis (AIH) and primary biliary cholangitis (PBC) variant syndrome (AIH-PBC VS) has not been achieved so far and evidence-based recommendations for monitoring and treatment of the disease are still lacking. Our study aimed to assess the prevalence, biochemical, and serological features, as well as the clinical course, of VS. Methods: We performed a retrospective study including all patients with VS between 1999 and 2020 in four German centers. Data on demographic parameters, biochemical and serological tests, treatment, and outcome were collected. Results: Of 90 patients (3.1%) meeting Paris criteria for VS diagnosis, 65.6% showed AIH and PBC histological features, while biochemical Paris criteria were observed comparatively rarely. Further antibodies, which were not part of the diagnostic criteria of VS, were found in a subgroup of patients with available data (ACA: 30.0%; anti-CENP-A: 25.0%; anti-CENP-B: 33.3%; anti-SP100: 21.4%). Biochemical response was more frequently observed in patients treated with a combined therapy of ursodeoxycholic acid (UDCA) and immunosuppression (IS). Liver cirrhosis was detected in 31 patients (34.4%) and 25 patients (27.8%) developed clinical manifestations of portal hypertension. Conclusions: Biochemical Paris criteria of VS were rarely detected, thus implying that these cut-off values should be redefined. Regarding pharmacological treatment, combined therapy of UDCA and IS appeared to be more effective than monotherapy with UDCA.

## 1. Introduction

Autoimmune liver diseases (AILD) comprise a group of immune-mediated liver diseases that include autoimmune hepatitis (AIH), primary biliary cholangitis (PBC), and primary sclerosing cholangitis (PSC) [1]. Each of them are distinct entities, although they share some similarities clinicopathologically [2]. Whether the coexistence of PBC and AIH is sequential, a concurrent occurrence of two independent autoimmune liver diseases (AILDs), or a primary AILD with one or more features of another AILD, is still under debate [1,3,4]. Furthermore, the diagnosis of variant syndrome (VS) remains a diagnostic challenge as autoimmune liver diseases show considerable heterogeneity and the boundaries between different entities are not well delineated. For the diagnosis of VS in PBC, several criteria have been established: non-response to ursodeoxycholic acid (UDCA), presence of AIH-specific autoantibodies, a remarkable elevation of transaminases and immunoglobulin G (IgG) levels, and a modified hepatic activity index (mHAI) score over 4/18 are such indicators for a VS of PBC [5,6]. However, an international consensus and standardized diagnostic criteria are lacking. The European Association of the Liver (EASL) statement on the “Paris criteria” emphasizes the importance of histology, but it is still uncertain whether other diagnostic criteria such as autoantibodies, alanine aminotransferase (ALT) elevation, and IgG should be given equal weight in this evaluation. Besides that, the specificity and accuracy of antibodies that are prognostic for classical AIH such as anti-smooth muscle antibodies (anti-SMAs) or antibodies to soluble liver antigen/liver-pancreas antigen (anti-SLA/LP) for diagnosing VS are still not clear and need to be investigated [7,8].

Due to the absence of a diagnostic consensus definition, the true prevalence of VS remains obscure, with different study results ranging from 2% to 20% [3]. Moreover, diagnosing a VS appears to be of clinical importance, because an unfavorable course of disease with progressing fibrosis and increased liver-related mortality compared to AIH or PBC alone has been reported [9,10,11]. Compared to the PBC group, patients with PBC and AIH overlap have shown higher rates of liver-related complications such as symptomatic portal hypertension (PHT), esophageal varices, gastrointestinal bleeding, or ascites [10,12]. Due to the low prevalence of VS, it is also not clear whether combined treatment of UDCA and immunosuppressive (IS) agents is superior to monotherapy with UDCA in these patients.

In summary, the diagnostic criteria for AIH-PBC VS have not been standardized, nor has the frequency of the disease been established. Management of AIH-PBC VS is still a clinical challenge, since it remains poorly characterized in many ways and the current knowledge is mainly based on clinical experience and retrospective case studies. The goal of the current study was to better characterize clinical characteristics and the clinical course of PBC-AIH VS by comparing the disease entity with control cohorts of AIH and PBC. Beyond that, we aimed to evaluate the available treatment options for AIH-PBC VS.

## 2. Materials and Methods

### 2.1. Study Population and Diagnostic Criteria

In this retrospective study, we aimed to compare a study cohort consisting of AIH-PBC VS patients with control cohorts of patients with AIH and of patients with PBC. Therefore, adult patients aged 18 and over with a PBC-AIH VS, AIH, or PBC, who attended the participating hospitals (University Hospital Frankfurt, University Hospital LMU Munich, University Medical Center Mainz, and University Hospital Heidelberg) between 23 August 1999 and 28 October 2020, were included in the study. Possible patients were identified by systematically searching the patient chart database of the participating hospitals for AIH- and PBC-related diagnosis codes K75.4 and K74.3 of the International Classification of Diseases, Tenth Revision (ICD-10), German Modification.

According to the Paris criteria, which have been endorsed by EASL, the diagnosis of AIH relied on the simultaneous or consecutive presence of at least two out of three biochemical, serological, and histological criteria of AIH. Diagnosis of PBC was made based on the simultaneous or consecutive presence of at least two out of three biochemical, serological, and histological criteria of PBC [13]. The PBC criteria included (I) alkaline phosphatase (ALP) levels at least two times or gamma glutamyltransferase (gGT) levels at least five times the upper limit of normal (ULN), (II) presence of AMA, and (III) a liver biopsy showing florid bile duct lesions. The AIH component of an overlapping syndrome was defined by (I) alanine transaminase (ALT) levels at least five times ULN, (II) IgG levels at least two times ULN or the presence of anti-SMAs, and (III) moderate or severe periportal and periseptal lymphocytic piecemeal necrosis.

According to the current EASL guidelines, the diagnosis of a PBC without VS was made based on fulfilment of at least two of three key criteria, namely a persistently elevated ALP over at least six months, the presence of serum AMA, and liver histology consistent with PBC [6].

AIH was defined based on simplified criteria (score > 6) proposed by the International Autoimmune Hepatitis Group (IAIHG) in 2008 [14].

Diagnosis of liver fibrosis and cirrhosis was at the physician’s discretion and was established by ultrasound, laboratory parameters, and non-invasive liver fibrosis assessments according to EASL guidelines [15].

In terms of biochemical response, incomplete response and remission were defined for the AIH component according to international guidelines [15,16]. Remission was defined as normalization of serum transaminases and IgG during treatment within a time frame of six months after initiation of treatment according to latest clinical practice guidelines and according to a recent consensus statement of the IAIHG [16]. Partial biochemical response was defined as normalization of either serum transaminases or IgG levels within six months after initiation of treatment. Biochemical non-response was defined by <50% decrease in serum transaminases within four weeks after treatment initiation. A biochemical response for the PBC component was defined according to Barcelona criteria as a >40% decrease in the pretreatment ALP levels or normalization one year after initiation of treatment [6]. Accordingly, inadequate response was defined by a decrease in ALP ≤ 40% and ALP ≥ 1 × ULN twelve months after initiation of treatment.

Initial management and type of treatment were at the physician’s discretion. Since the optimal type of treatment was not known, the managing physician was free to decide whether to treat first with UDCA monotherapy (13–15 mg/kg/day) or with combined therapy of UDCA and immunosuppressors (UDCA + IS; prednisolone 30–60 mg/day alone or in combination with azathioprine 50–150 mg/day). Non-responders to UDCA were considered for combined UDCA and immunosuppression, as second-line immunosuppressive agent mycophenolate mofetil (MMF) was used when standard immunosuppression failed to achieve remission.

According to the initial type of therapy, the patients were divided into two groups: the first group, which was treated with UDCA monotherapy, and the second group treated with UDCA + IS.

In order to evaluate the absolute risk of liver transplantation or death at baseline and one year after treatment initiation, the GLOBE Score was calculated at baseline for the PBC cohort and at baseline, pre- and posttreatment after one year of therapy for patients with AIH-PBC VS. The GLOBE Score contained patients’ age, levels of bilirubin, ALP, albumin, and platelet count. The following formula was applied: 0.044378 × age at baseline + 0.93982 × bilirubin (×ULN) + 0.335648 × ALP (×ULN) + 2.266708 × albumin (×Lower Limit of Normal (LLN)) + 0.002581 × platelets (per 10⁹/L) + 1.21685 [17].

### 2.2. Data Collection

Extracted patients’ characteristics included gender, age, and BMI at baseline. In addition, liver biochemical and serological tests, imaging study of the liver, histopathological results, clinical manifestation, medical therapies used, and liver-related complications were recorded at baseline and at yearly follow-up.

The available data for the various epidemiologic, clinical, virologic, and biochemical parameters were collected. Medians, ranges, and percentages were always calculated based on the corresponding available data, and are listed in Appendix A. A detailed description of methods applied for testing on autoantibodies is also provided in the Appendix A.

This study was approved by the local ethics committee of the Goethe University Frankfurt (vote 2021–481) and the involved partners. Patients were excluded if they were younger than 18 years old or pregnant. Owing to the retrospective, anonymous, and non-interventional nature of this study, no informed consent from individual patients had to be obtained.

### 2.3. Statistical Analysis

Statistical analyses were performed using IBM SPSS 26.0 statistical software package (SPSS/IBM, Munich, Germany). Characteristics of the cohort were examined by descriptive statistics (percentages, medians, standard deviation, etc.). Associations between categorial and continuous variables were tested by chi-square test and Mann–Whitney U Test, respectively. All tests were two-sided and a *p*-value of less than 0.05 was judged to be statistically significant.

## 3. Results

### 3.1. Baseline and Clinical Characteristics

The main baseline patient characteristics of the overall study population, including demographic and clinical features, are listed in Table 1.

**Figure 1:**
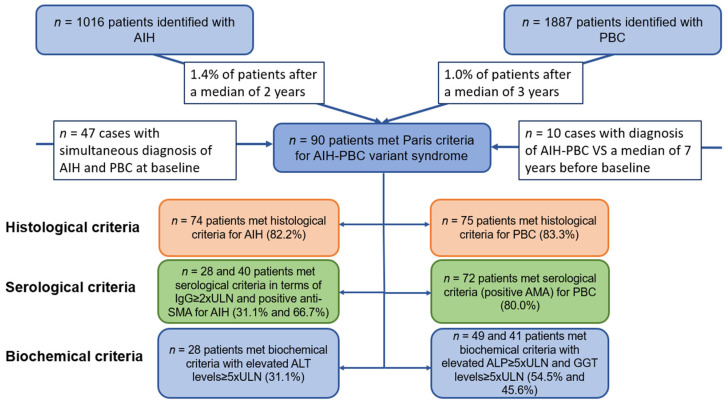
Overview of the study cohort and the percentage of patients fulfilling the Paris criteria. AMA = Antimitochondrial Antibodies; Anti-SMA = Anti-Smooth Muscle Antibodies.

A total of 90 patients with AIH-PBC VS were identified. Eighty patients (89%) were women with a median age of 49 years (range, 19–77). Follow-up was available in 84 of these patients. Median duration of follow-up from diagnosis of AIH-PBC VS to last follow-up was seven years (range, 0.5–21). Of 1016 patients with the initial diagnosis of AIH, 14 (1.4%) met the Paris criteria for diagnosing an AIH-PBC VS after a median of two years (range, 0.2–20), as shown in Figure 1.

Moreover, of 1887 patients with initial diagnosis of PBC, 19 (1.0%) satisfied the criteria of AIH-PBC VS after a median of three years (range, 0.25–11). In 47 cases (1.6%), simultaneous forms of AIH-PBC VS were diagnosed (Table 1). In 15 cases, diagnosis of AIH-PBC VS had already been confirmed a median of seven years before baseline.

At baseline, patients presented with abnormal liver parameters: elevated alkaline phosphatase (ALP) and gamma glutamyl transferase levels (GGT) were detected in 82.2% (*n* = 74) and 85.6% (*n* = 77) of patients. Elevated ALT and AST levels were observed in 81.1% (*n* = 73) and 78.9% (*n* = 71) of patients at baseline.

Cholestatic liver parameters as well as transaminase levels significantly decreased at the end of follow-up compared to baseline (ALP: *p* < 0.001; GGT: *p* < 0.001; AST: *p* < 0.001; ALT: *p* < 0.001).

Liver biopsy was available for all patients. Of these, 15 patients (16.7%) exclusively revealed typical histological features of AIH such as portal or periportal infiltration of lymphocytes and plasma cells with interface hepatitis (Table 1). Severe interface hepatitis was found in *n* = 20 of these patients (22.2%). In a further 16 patients (17.8%), exclusively biliary changes typical of PBC, such as destructive cholangitis or ductopenia, were found, whereas 59 (65.6%) of patients revealed histological features of both AIH and PBC.

Concerning the presence of diagnostic criteria, histological and serological findings of PBC with positive AMA-antibodies were observed to be frequent among patients with VS (83.3% and 80.0%, respectively). In contrast, especially biochemical criteria (elevated ALP and GGT levels) were found less frequently (54.5% and 45.6%, respectively). Accordingly, only 31.1% of patients fulfilled the biochemical criterion of an AIH component with elevated ALT levels at least five times the ULN. Comparably, serological criteria of IgG at least two times the ULN and of positive anti-SMA antibodies were only found in 31.1% and 66.7% of patients, whereas histological findings of AIH were detected more frequently (82.2%). IgG levels above the ULN were observed in 65% of patients.

To compare the clinical criteria and clinical course of the overlap cohort, control cohorts of *n* = 100 patients with AIH and *n* = 100 patients with PBC were recruited. The diagnosis of AIH or PBC was confirmed histologically in all of these patients.

A total of 91% of the PBC controls fulfilled serological diagnostic criteria with positive AMAs, while 72% met biochemical diagnostic criteria with elevated ALP values.

As serological criteria of the simplified IAIHG score, ANA, SMA, LKM-1, and SLA were found in 83%, 34%, 14%, and 17% of the included AIH controls, respectively. In addition, elevated levels of IgG were detected in 57% of these patients. Serum biochemistry, including ALP, total bilirubin, and GGT, was not significantly different between PBC, AIH, and the AIH-PBC VS cohort. In contrast, significantly elevated serum ALT and AST levels were observed in patients with AIH-PBC VS compared to PBC patients (*p* = 0.04, *p* = 0.03). In addition, however, AIH controls had significantly higher AST and ALT levels than patients with AIH-PBC VS (*p* = 0.04, *p* = 0.02).

Regarding liver-related outcomes, a total of 34 patients (37.8%) of the overlap cohort presented with clinically advanced hepatic involvement at diagnosis; at baseline, significant fibrosis and cirrhosis was detected in 18 and 16 patients (20.0% and 17.8%), respectively. During follow-up of the study, a further seven patients (7.8%) developed significant fibrosis a median of three (range, 0–9) years after diagnosing AIH-PBC VS, while a further 15 patients (16.7%) presented with liver cirrhosis a median of six (range, 0–11) years after diagnosis of AIH-PBC VS. Clinical manifestations of portal hypertension such as hydropic decompensation and esophageal varices were detected in ten (11.1%) and seven (7.8%) of these patients during the observation period, respectively. Moreover, portal vein thrombosis was found in four patients, three patients received transjugular intrahepatic portosystemic shunt (TIPS) for refractory ascites or variceal bleeding, and one patient (1.1%) underwent liver transplantation during follow-up. Hepatocellular carcinoma was diagnosed in one patient (1.1%) and a further two patients (2.2%) died during follow-up due to liver-related complications.

When comparing liver-related outcomes in the overlap cohort with the corresponding control cohorts, patients with AIH-PBC VS were more likely to suffer from advanced stages of liver disease both at baseline and during study follow-up. Liver cirrhosis and significant fibrosis were observed to be significantly more common in AIH-PBC VS patients compared to AIH controls at both baseline and end of follow-up (liver cirrhosis at baseline in AIH-PBC VS vs. AIH controls: *n* = 16 vs. *n* = 5, *p* = 0.006; significant fibrosis at baseline in AIH-PBC VS vs. AIH controls: *n* = 18 vs. *n* = 8, *p* = 0.02; liver cirrhosis at the end of follow-up in AIH-PBC VS vs. controls: *n* = 31 vs. *n* = 10, *p* < 0.001; and significant fibrosis at the end of follow-up in AIH-PBC VS vs. AIH controls: *n* = 25 vs. *n* = 13, *p* = 0.02). Accordingly, a trend toward a higher frequency of significant fibrosis and cirrhosis was observed in patients with AIH-PBC VS compared to PBC controls at baseline and throughout the study (liver cirrhosis at baseline in AIH-PBC VS vs. PBC controls: *n* = 16 vs. *n* = 11, *p* = 0.21; significant fibrosis at baseline in AIH-PBC VS vs. PBC controls: *n* = 18 vs. *n* = 13, *p* = 0.24; liver cirrhosis at the end of follow-up in AIH-PBC VS vs. PBC controls: *n* = 31 vs. *n* = 17, *p* = 0.007; and significant fibrosis at the end of follow-up in AIH-PBC VS vs. PBC controls: *n* = 25 vs. *n* = 17, *p* = 0.08). Beyond that, we compared liver-related adverse events, including the incidence of complications of portal hypertension, liver-related mortality, and liver transplantation between the cohorts. Patients with AIH-PBC VS (*n* = 25) more frequently suffered from complications of portal hypertension compared to PBC (*n* = 13) and AIH controls (*n* = 7), respectively (*p* = 0.02; *p* < 0.001). Moreover, liver-related mortality and the rate of liver transplantation were insignificantly higher in patients with AIH-PBC VS (*n* = 2; *n* = 1) compared to AIH (*n* = 0; *n* = 0; *p* = 0.22; *p* = 0.47) and PBC controls (*n* = 1; *n* = 0; *p* = 0.60; *p* = 0.47).

Comparing median GLOBE Scores of the AIH-PBC VS cohort with those of PBC controls, significantly higher values were observed for patients with AIH-PBC VS at baseline (AIH-PBC VS: median −3.56 (range, (−8.99)–(−2.66)) vs. −6.24 (range, (−9.44)–(4.29); *p* < 0.001).

### 3.2. Serological Findings

Serological features of the study population are presented in Table 2.

ANA and anti-SMAs, both common in type 1 AIH, were detected in 71.4% and 66.7% of patients with AIH PBC VS and available information, respectively. Anti-liver-kidney-microsomal type 1 antibodies (anti-LKM-1), which are the serological hallmark of type 2 AIH, were positive in 3.6% of AIH-PBC patients with available data.

In addition, serologic analyses for the presence of anti-soluble liver antigen/liver-pancreas antibodies (anti-SL-A/LP) were performed in 62 cases, of which five were positive (8.1%). Concerning further antibodies associated with AIH, anti-LC-1 and pANCA were detected in 5.9% and 20% of patients with available information, respectively. ANCA directed at the key antigenic targets PR-3 and MPO were found in 18.2% and 15.4% of patients.

Regarding non-specific subtypes of ANA, anti-Ro/SSA, ACA, anti-CENP-A, anti-CENP-B, anti-gp210, and anti-Sp100 were those detected frequently among patients with available information (anti-Ro/SSA: 17.6%, ACA: 30.0%; anti-CENP-A: 25.0%; anti-CENP-B: 33.3%; anti-gp210: 13.3%; anti-SP100: 21.4%).

In contrast, other ANA subtypes such as anti-dsDNA, anti-Scl 70, U1-RNP, Sm-RNP, anti-La/SSB, and anti-PML were not detected or were detected only in a minority of patients (Table 2).

Antimitochondrial antibodies (AMAs), which are considered the serological hallmark of PBC, were found in 72 out of 90 tested patients (80.0%), with AIH-PBC VS. AMA-M2 and AMA M2-3E antibodies, which are highly specific for PBC, detected in 72.2% and 70% of tested patients, respectively.

Serologically, PBC patients in the control cohort were most frequently positive for AMA antibodies (91.0%), followed by less frequently positive PBC-specific ANA antibodies anti-Gp210 and anti-sp100 (25.0% and 33.3%, respectively). Additionally, positive antibodies against PML as well as dsDNA were detected in 46.7 and 22.2% of the PBC patients.

Most commonly detected antibodies in the AIH control cohort were ANA positive antibodies (83.0%), followed by anti-PR 3 and anti-SMA (37.5% and 33.8%).

### 3.3. Response to Therapy

Information on treatment type, as well as biochemical and clinical response, was available for 84 patients. Of these, 21 patients were initially treated with UDCA alone at 13 to 15 mg/kg/d, of whom 14 patients subsequently received a combination of UDCA and immunosuppressive agents due to lacking biochemical response. A total of 63 patients received a combination of UDCA and standard immunosuppression as first-line therapy. Second-line immunosuppressive treatment was initiated in six patients, who did not respond to initial immunosuppression. The remission rate at the end-of-follow up was significantly higher in the combined group than in the UDCA group (76.2% vs. 42.9%; *p* = 0.01).

#### 3.3.1. Outcome of Patients Treated with UDCA Alone (*n* = 21)

A total of 21 patients were treated with UDCA alone for a median of five years (range, 1–30). AP, ALT, bilirubin, and GGT levels decreased under monotherapy with UDCA. However, only the biochemical trend of GGT values was observed to be significant (*p* = 0.01).

Complete biochemical response was observed in four of these patients a median of 5.3 months (range, 2–6) after initiation of treatment (19.0%).

A total of twelve further patients (57.1%) showed a partial response to UDCA monotherapy. Normalization of liver tests but not IgG was only observed in *n* = 3 patients, whereas normalization of IgG but not of liver parameters was detected in *n* = 9 patients. Biochemical non-response was detected in *n* = 5 patients (23.8%). None of the complete responders had severe interface hepatitis on histology. In contrast, it was frequently observed in patients with biochemical partial and non-response (Table 3).

In most partial responders (*n* = 4) and non-responders (*n* = 5), biochemical parameters of cholestasis decreased with UDCA monotherapy, but serum aminotransferases did not normalize but remained stable or increased. Moreover, *n* = 4 of the UDCA partial responders and *n* = 3 of the non-responders experienced hepatitic flares with an increase in aminotransferases under UDCA monotherapy. Additional immunosuppression was administered in 14 patients a median of 4.75 years (range, 1–12) after therapy initiation of UDCA.

Regarding the clinical course of the disease, two UDCA responders had cirrhosis and one patient had significant liver fibrosis at baseline. None of the patients experienced disease progression during treatment.

Regarding patients with partial biochemical response, two patients had significant liver fibrosis and three patients suffered from liver cirrhosis at the onset of therapy. Progression of disease was observed in three patients: two patients without relevant fibrosis and one patient with significant fibrosis at baseline developed manifest liver cirrhosis during follow-up. Among the UDCA non-responders, three patients suffered from liver cirrhosis at baseline. Two of these patients experienced progression of disease and worsening of portal hypertension during therapy (median MELD at onset of therapy: 11 (range, 9–13); median MELD at end of follow-up: 15 (range, 11–18)), one of whom was transplanted a median of 15 years after initial diagnosis of VS. Moreover, two further biochemical non-responders to UDCA monotherapy had significant liver fibrosis at baseline and developed liver cirrhosis a median of 3.5 years later.

Median GLOBE Score before introducing UDCA (*n* = 21) and one year post-treatment decreased from −3.71 (range, (−6.78)–(2.66)) to −4.92 (range, (−7.91)–(3.42); *p* = 0.18).

#### 3.3.2. Outcome of the IS + UDCA Group (*n* = 63)

The combination of UDCA and corticosteroids was administered to 63 patients for a median of seven years (range, 0.6–23). In 44 cases, azathioprine (AZA) was added as a corticosteroid sparing agent. Complete biochemical response could be detected in 37 patients (58.7%) a median of 5.7 months (range, 3.4–6.1) after initiation of treatment. Among the 26 other patients, 18 (28.6%) had a partial biochemical response and non-response was detected in *n* = 8 patients (12.7%). In six cases, a second-line therapy with mycophenolate mofetil (MMF) was initiated; four of these patients had an inadequate biochemical response to AZA and two patients had an AZA intolerance.

Comparing biochemical responders with partial responders and non-responders, a significantly higher frequency of severe interface hepatitis was detected in non-responders (*p* = 0.02) (Table 4).

In the group of biochemical responders, eleven patients had significant fibrosis at baseline, and seven of these patients had stable disease during therapy. However, four patients experienced progression of fibrosis to liver cirrhosis by the end of treatment. Five additional patients with complete biochemical response during follow-up had liver cirrhosis at baseline: four of them developed complications of portal hypertension and median MELD increased from 8 (range, 6–10) to 13 (range, 11–15) during the course of treatment.

Regarding patients with partial biochemical response, three had significant liver fibrosis at baseline, all of whom developed liver cirrhosis during follow-up. Moreover, one out of two patients with liver cirrhosis at baseline and incomplete biochemical response suffered from complications of portal hypertension during follow-up.

Of the patients who did not biochemically respond to UDCA + IS, one had cirrhosis at baseline and experienced disease progression. No significant fibrosis was detected at baseline in the non-responder group. However, three patients with no tissue damage at baseline and no biochemical response developed significant fibrosis and cirrhosis at a median of five years (range, 4–6).

Regarding GLOBE Scores at pretreatment and one year after treatment with UDCA + IS, values significantly decreased from −3.23 to –6.28 (range, (−7.91)–(3.43) *p* = 0.04). Furthermore, when comparing GLOBE scores one year after UDCA monotherapy with those after one year of UDCA + IS combination therapy, insignificantly higher scores were observed in patients treated with UDCA monotherapy (median GLOBE score −4.92 vs. −6.28; *p* = 0.08).

## 4. Discussion

In our study, we used the strict Paris criteria to define an AIH-PBC VS. The resulting prevalence of AIH-PBC VS in our 1016 AIH and 1887 PBC patients was low at 4.0%, which is consistent with previous studies using the Paris criteria [18,19,20]. However, the literature on the prevalence of AIH-PBC VS is inconclusive, and overall prevalence rates in published reports range from 2% to 27% [11,12,21,22,23,24]. We assume that this observed wide range of prevalence rates might be primarily due to differing diagnostic criteria used. In the absence of standardized diagnostic criteria, several definitions of VS exist and distinct diagnostic approaches have been applied in the literature. Besides the most commonly used Paris criteria, which have been endorsed by the EASL, revised and simplified IAIHG scoring systems have also been used to diagnose VS of PBC in the past. Prevalence rates in studies using these scoring systems to diagnose AIH in PBC patients were much higher, ranging from 6 to 27% [11,22]. Nevertheless, these scores were not intended for diagnosing AIH-PBC VS and the IAIHG advises against their application in this setting [23].

The EASL statement on the “Paris criteria” emphasizes that histological evidence of interface hepatitis is mandatory to diagnose an VS [23]. However, it is not entirely clear whether other diagnostic criteria such as autoantibodies or elevated ALT and IgG should be given equal weight in this score. Histological features of AIH and PBC were found in 59 (65.6%) of patients with VS, whereas biochemical markers were detected comparatively rarely. Elevated ALT levels of at least five times the ULN were found in only 31.1% of patients, indicating that this biochemical cut-off may be rather high and should be further validated. The intention of choosing these high cut-off values may be to avoid overdiagnosis and unnecessary use of immunosuppressants to treat elevated transaminases in classical PBC patients, which may be due to cholestasis-mediated hepatocyte injury. On the other hand, this relatively high cut-off may be responsible for missing diagnoses of AIH-PBC VS in patients who would benefit from immunosuppression. Since liver biopsy is considered the gold standard for assessing the degree of hepatitis, and AIH-positive histology was found in the majority of our AIH-PBC VS patients, a lower threshold of transaminases may be useful to indicate the performance of a liver biopsy.

Beyond positive liver histology and elevated transaminases, the Paris criteria support the diagnostic value of anti-SMAs for diagnosing an VS of PBC [19]. In our study, anti-SMAs were detected in two-thirds of patients with VS (66.7%). However, anti-SMAs are less specific for AIH and are also detected in up to 20% of PBC patients [25]. Thus, the presence of anti-SMAs may not always prove the coexistence of AIH in PBC patients, but might also reflect classical PBC with hepatitic features.

As another part of the Paris criteria for the diagnosis of PBC in VS, AMA and AMA-M2 were frequently found in the subcohort of patients tested. Besides that, detection of further antibodies such as ANA subtypes (ACA, anti-gp210, anti-Sp100, anti-CENP-A, and anti-CENP-B) was observed in the subcohort of patients tested on these antibodies.

It is probable that all of these tested antibodies have a greater relevance to the diagnostic value of a variant syndrome, as well as to the prognosis and natural course of the disease. However, the limited number of patients tested and the retrospective nature of the study make it impossible to draw further conclusions, and prospective studies are needed to investigate this further.

Due to the absence of standardized diagnostic criteria for AIH-PBC VS and the relatively low prevalence of the disease, randomized controlled clinical trials are lacking and current treatment recommendations are mainly based on retrospective case studies and clinical experience. Consistent with previous results, our study showed that treatment with UDCA alone was not effective in the majority of patients with VS: only 19% achieved a complete biochemical response with UDCA monotherapy. Moreover, the fact that none of these patients with complete biochemical response to UDCA monotherapy had severe interface hepatitis on histology might indicate that only patients with mild to moderate AIH activity might benefit from UDCA monotherapy and others should be treated with add-on administration of immunosuppression. Moreover, the high rate of patients experiencing progression of disease among partial responders and non-responders to UDCA monotherapy demonstrates that immunosuppression should be evaluated promptly in cases of an insufficient biochemical response to UDCA.

In contrast, complete biochemical response was achieved in 58.7% of patients treated with UDCA and additional IS. Moreover, a relatively high proportion of patients with biochemical response to UDCA and IS experienced a stable course of disease and stabilization of liver disease: 7 out of 11 patients experienced stability or decrease of fibrosis, and among patients without any tissue damage at baseline (*n* = 21), none of them developed fibrosis during follow-up of the study. Beyond that, the decrease in median GLOBE Scores was more pronounced in patients treated with UDCA + IS combination therapy compared to UDCA monotherapy. This observed benefit of combined UDCA and immunosuppressive treatment in patients with VS has been confirmed by several other studies [26,27]. However, despite biochemical response, disease progression was also observed in patients treated with UDCA + IS at the end of follow-up compared to baseline, and the total number of patients with fibrosis and cirrhosis increased at the end of treatment, suggesting that, despite biochemical normalization of liver enzymes, complete remission of histological inflammatory activity cannot be assumed in all patients. Similar observations on persistent histological activity of AIH despite complete biochemical response have been made previously [28]. Thus, especially in cases of progressive liver disease despite biochemical response, histological assessment should be evaluated in order to determine treatment response.

Data on the natural history and long-term outcomes of patients with AIH-PBC VS are also limited and conflicting. Joshi et al. reported a comparable survival of patients with VS and patients with classical PBC [20]. In contrast, a worse clinical course of VS with increased liver-related mortality and faster progression of fibrosis has been observed by others [10,11]. Consistent with these observations, in our analysis patients with VS had a worse prognosis than the corresponding control cohorts. Patients with VS had a higher incidence of progression to cirrhosis and complications of portal hypertension. Moreover, GLOBE Scores were observed to be considerably higher in patients with VS at baseline compared to the PBC cohort, demonstrating that these patients have a diminished LT-free survival. These observations underscore the importance of early and correct diagnosis and careful monitoring of patients with AIH-PBC VS.

A major limitation of our study is its retrospective design, which requires careful interpretation of the results. Diagnostic tests such as biochemical values, as well as initiation or change in therapeutic regimens, were at the physician’s discretion and not inspired by a predefined study protocol. Results of the study might be influenced by selection and surveillance bias. Therefore, conducting a prospective study with extended follow-up is very difficult.

## 5. Conclusions

In conclusion, our multicenter, retrospective cohort emphasizes the importance of correct and early identification of AIH-PBC VS, as an unfavorable disease course with rapid progression to fibrosis and increased liver-related mortality was observed.

Diagnostic results showed that histological features of AIH and PBC were frequently found in patients with VS, whereas biochemical parameters of Paris criteria were detected comparatively rarely. The rather high biochemical cut-off levels might be responsible for missing diagnoses of AIH-PBC VS in patients, who could benefit from immunosuppression, and should be validated further.

In addition, our results showed the occurrence of antibodies such as pANCA, anti-CENP-A, anti-CENP-B, ACA, and anti-sp100, which are not part of the diagnostic work-up of AIH-PBC VS. It is probable that the determination of these antibodies might help to diagnose AIH-PBC VS in the future. However, prospective studies with larger case numbers are needed to evaluate this further. Regarding pharmacological treatment options, combined therapy of UDCA and immunosuppression appeared to be more effective than monotherapy with UDCA. Nevertheless, future prospective studies are also warranted to evaluate the diagnostic and clinical relevance of the described antibodies in AIH-PBC VS and to analyze the effect of therapy, especially on clinically relevant endpoints such as progression to liver cirrhosis or liver-related death.

## Figures and Tables

**Table 1:** Baseline characteristics of patients at diagnosis of AIH-PBC variant syndrome and control cohorts.

Characteristics	AIH-PBC Cohort (*n* = 90)	AIH Control Cohort (*n* = 100)	PBC Control Cohort (*n* = 100)
Patient age at diagnosis (years), median (range)	49 (19–77)	64 (25–86)	76 (47–96)
Male/female, *n* (%)	10 (11)/80 (89)	29 (29)/71 (71)	0 (0)/100 (100)
Caucasian, *n* (%)	67 (74)	93 (93)	88 (88)
BMI (kg/m^2^), median (range)	26 (19–40)	23 (17.7–28.3)	22 (17.6–27.7)
Length of follow-up (years), median (range)	7 (0.5–21)	7 (0–20)	8 (0–19)
Liver biochemical tests, *n* (%)			
ALT level, ×ULN	2.9 (1.1–55.7)	6.1 (0.3–50.3)	1 (0.2–11.2)
AST level, ×ULN	4.2 (1.2–51.1)	8.1 (0.5–85.0)	1 (0.2–16.6)
ALP level, ×ULN	1.4 (0.3–6.3)	1.2 (0.3–8.0)	1.5 (0.5–6.1)
GGT level, ×ULN	3.5 (0.2–19.6)	2.3 (0.2–18.1)	1.5 (0.2–18.8)
Bilirubin level, ×ULN	0.6 (0.2–18.6)	0.9 (0.1–20.6)	0.4 (0.1–5.6)
Albumin level, ×LLN	0.9 (0.8–1.8)	0.9 (0.7–1.5)	0.8 (0.7–1.9)
IgM level, ×ULN	1.3 (0–3.9)	0.6 (0.1–1.6)	1.2 (0.2–2.9)
IgG level, ×ULN	1 (0.3–2.3)	1.1 (0.4–2.9)	0.7 (0.5–1.4)
Histological features			
of AIH *	15 (16.7)	100 (100)	
of PBC **	16 (17.8)		
of AIH and PBC	59 (65.6)		100 (100)
Clinical manifestation of autoimmune diseases			
Initial manifestation of AIH	14 (15.6)	100 (100)	
Initial manifestation of PBC	19 (21.1)		100 (100)
Simultaneous manifestation of AIH and PBC	47 (52.2)		
n. a.	10 (11.1)		
Significant liver fibrosis, *n* (%)	18 (20.0)	8 (8.0)	13 (13.0)
Liver cirrhosis, *n* (%)	16 (17.8)	5 (5.0)	11 (11.0)
MELD, median (range)	10 (5–23)	11 (11–27)	10 (6–20)
Child class A/B/C, *n* (%)	9 (18.9)/6 (6.7)/1 (1.1)	3 (3.0)/1 (1.0)/1 (1.0)	7 (7.0)/3 (3.0)/1 (1.0)
GLOBE Score pretreatment, median (range)	−3.56 (−8.99–2.66)		−6.24 (−9.44–4.29)

* moderate or severe or severe periportal or periseptal lymphocytic piecemeal necrosis. ** florid bile duct lesions. AIH = Autoimmune Hepatitis; ALP = Alkaline Phosphatase; ALT = Alanine Aminotransferase; AST = Aspartate Aminotransferase; BMI = Body Mass Index; GGT = Gamma Glutamyltransferase; IgG = Immunoglobulin G; IgM = Immunoglobulin M; LLN = Lower Limit of Normal; MELD = Model for End-Stage Liver Disease; PBC = Primary Biliary Cirrhosis; ULN = Upper Limit of Normal.

**Table 2:** Serological features of patients with AIH-PBC overlap syndrome and control cohorts.

Characteristics	AIH-PBC Cohort (*n* = 90)	AIH Control Cohort (*n* = 100)	PBC Control Cohort (*n* = 100)
ANA, *n* (%)	60/84 (71.4)	83/100 (83)	23/95 (24.2)
Non-specific subtypes of ANA			
Anti-Ro/SSA	3/17 (17.6)	4/22 (18.2)	3/15 (15.8)
SSA 52-60	4/14 (28.6)	5/20 (25.0)	3/16 (18.8)
Anti-La/SSB	0/14 (0)	4/18 (22.2)	1/17 (5.9)
Anti-Scl 70	0/16 (0)	0/8 (0)	0/10 (0)
ACA	3/10 (30.0)	1/21 (4.7)	2/16 (12.5)
Anti-gp210	2/15 (13.3)	0/12 (0)	3/12 (25.0)
Anti-Sp100	3/14 (21.4)	1/12 (8.3)	4/12 (33.3)
Anti-CENP-A	2/8 (25.0)	0/13 (0)	1/17 (5.9)
Anti-CENP-B	4/12 (33.3)	0/17 (0)	2/17 (11.8)
Anti-dsDNA	3/27 (11.1)	6/40 (15.0)	8/36 (22.2)
U1-RNP	0/9 (0)	0/8 (0)	0/10 (0)
Sm-RNP	0/15 (0)	1/8 (12.5)	0/6 (0)
Anti-PML	2/14 (14.3)	0/14 (0)	7/15 (46.7)
ANCA			
pANCA	4/20 (20.0)	3/30 (10.0)	0/32 (0)
Anti-MPO	2/13 (15.4)	3/29 (10.3)	0/32 (0)
Anti-PR 3	2/11 (18.2)	3/8 (37.5)	0/15 (0)
AMA	72/90 (80.0)	6/100 (6.0)	91/100 (91.0)
AMA-M2	26/36 (72.2)	1/16 (6.3)	8/36 (22.2)
AMA-M2-3E	14/20 (70.0)	0/8 (0)	6/14 (42.9)
Anti-SMA	40/60 (66.7)	24/71 (33.8)	0/32 (0)
Anti-LKM-1	2/55 (3.6)	9/63 (14.3)	0/32 (0)
Anti-LC1	1/17 (5.9)	0/31 (0)	0/35 (0)
Anti-SLA/LP	5/62 (8.1)	12/68 (17.6)	0/38 (0)
Anti-Jo1	0/17 (0)	0/12 (0)	0/29 (0)

ACA = Anticentromere Antibodies; AMA-M2 = Antimitochondrial Antibodies M2 subtype; AMA M2-3E = Antimitochondrial Antibodies M2-3E subtype; ANA = Antinuclear Antibodies; ANCA = Antineutrophil Cytoplasmic Antibodies; Anti-CENP-A = Antibodies against centromere protein A; Anti-CENP-B = Antibodies against centromere protein B; Anti-dsDNA = Antibodies against double-stranded DNA; Anti-gp210 = Antibodies against glycoprotein 210; Anti-Jo1 = Anti-Histidyl-tRNA Synthetase; Anti-La/SSB = Antibodies against La/SSB antigen; Anti-LC1 = Anti-Liver Cytosol Antibody Type 1; Anti-LKM-1 = Anti-Liver-Kidney-Microsomal Type 1 Antibodies; Anti-MPO = Anti-Myeloperoxidase; Anti-PML = Antibodies against promyelocytic leukemia protein; Anti-PR 3 = Anti-Proteinase 3; Anti-SLA/LP = Anti-Soluble Liver Antigen/ Liver Pancreas; Anti-Ro/SSA = Antibodies against Ro/SSA antigens; Anti-Scl 70 = Antibodies against Scl-70 antigen; Anti-Sp100 = Antibodies against Sp100 protein; pANCA = Perinuclear Antineutrophil Cytoplasmic Antibodies; Sm-RNP = Antibodies against Smith antigen and ribonucleoprotein; SSA 52-60 = Antibodies against RO/SSA 52/60; U1-RNP = Antibodies against U1 ribonucleoprotein.

**Table 3:** Biochemical and clinical response to different treatment regimens.

Parameters	UDCA Responders (*n* = 4)	UDCA Partial Responders (*n* = 12)	*p*	UDCA Responders (*n* = 4)	UDCA Non-Responders (*n* = 5)	*p*
Female gender	4/4 (100)	11/12 (92)	1.00	4/4 (100)	4/5 (80)	1.00
Age	46 (29–74)	51 (38–69)	0.60	46 (29–74)	54 (33–72)	0.56
ALT × ULN	1.5 (0.8–2.1)	1.7 (1.4–9)	0.26	1.5 (0.8–2.1)	3.0 (1.5–22)	0.09
AST × ULN	1.4 (0.7–3.2)	2.3 (0.8–4.3)	0.36	1.4 (0.7–3.2)	3.8 (0.9–12)	0.11
ALP × ULN	1.1 (0.5–2.8)	1.5 (1.3–4.7)	0.26	1.1 (0.5–3.1)	1.4 (1.8–3.5)	0.42
GGT × ULN	2.2 (0.4–4.3)	3.0 (1.9–13)	0.38	2.2 (0.4–4.3)	3.5 (1.0–19)	0.29
IgG × ULN	0.8 (0.6–1.1)	0.9 (0.7–1.1)	0.90	0.8 (0.6–1.1)	1.2 (0.7–1.5)	0.11
IgM × ULN	0.9 (0.2–3.9)	1.3 (0.4–3.5)	0.87	0.9 (0.2–3.9)	1.4 (0.5–4.1)	0.56
ANA	3/4 (60)	7/10 (70)	1.00	3/4 (60)	3/5 (60)	1.00
SMA	3/4 (75)	7/9 (67)	1.00	3/4 (75)	3/4 (75)	1.00
AMA	4/4 (100)	9/12 (75)	0.53	4/4 (100)	4/5 (80)	1.00
AMA-M2	2/3 (67)	4/6 (66.7)	1.00	2/3 (67)	1/2 (50)	1.00
Significant fibrosis	1/4 (25)	2/12 (17)	1.00	1/4 (25)	1/5 (20)	1.00
Liver cirrhosis	2/4 (50)	3/12 (25)	0.55	2/4 (50)	3/5 (60)	1.00
Severe histological interface hepatitis	0/4 (0)	4/12 (33)	0.52	0/4 (0)	3/5 (40)	0.17

**Table 4:** General characteristics of patients treated by UDCA + IS (*n* = 63).

Parameters	Therapy Responders (*n* = 37)	Partial Responders (*n* = 18)	*p*	Therapy Responders (*n* = 37)	Therapy Non-Responders (*n* = 8)	*p*
Female gender	31/37 (84)	16/18 (89)	1.00	31/37 (84)	6/8 (75)	0.62
Age	51 (26–72)	50 (26–77)	0.82	51 (26–72)	53 (18–72)	0.61
ALT × ULN	4.6 (0.2–41.3)	4.2 (0.3–12.6)	0.18	4.6 (0.2–41.3)	5.1 (0.4–15.9)	0.53
AST × ULN	4.4 (0.5–36.8)	4.1 (0.6–11)	0.19	4.4 (0.5–36.8)	5.0 (0.4–17.9)	0.63
ALP × ULN	1.1 (0.4–5.1)	1.6 (0.3–6.3)	0.34	1.1 (0.4–5.1)	1.2 (0.6–4.6)	0.80
GGT × ULN	3.3 (0.3–12.1)	3.6 (0.7–16.4)	0.45	3.3 (0.3–12.1)	2.2 (0.2–5.5)	0.16
IgG × ULN	0.9 (0.4–1.9)	0.8 (0.4–2.3)	0.93	0.9 (0.4–1.9)	1.1 (0.8–1.5)	0.37
IgM × ULN	1.2 (0.4–2.6)	1.3 (0.5–3.2)	0.39	1.2 (0.4–2.6)	1.8 (0.7–3.1)	0.07
ANA	24/35 (69)	12/17 (71)	1.00	24/35 (69)	6/8 (75)	1.00
SMA	17/25 (68)	7/11 (64)	1.00	17/25 (68)	4/6 (67)	1.00
AMA	30/37 (81)	14/18 (78)	1.00	30/37 (81)	6/8 (75)	0.65
AMA-M2	10/13 (77)	6/8 (75)	1.00	10/13 (77)	3/4 (75)	1.00
Significant fibrosis	11/37 (30)	3/18 (17)	0.35	11/37 (30)	0/8 (0)	0.17
Liver cirrhosis	5/37 (11)	2/18 (17)	1.00	5/37 (11)	1/8 (13)	1.00
Severe histological interface hepatitis	4/37 (11)	6/18 (33)	0.06	4/37 (11)	4/8 (50)	0.02

## Data Availability

The data that support the findings of this study are listed in Table 1, Table 2, Table 3 and Table 4.

## Appendix A

### Appendix A.1. Material and Methods

#### Appendix A.1.1. Data Collection

Gender, *n* = 90; age, *n* = 90; ethnicity, *n* = 89; BMI, *n* = 89; liver fibrosis, *n* = 90; liver cirrhosis, *n* = 90; alanine aminotransferase (ALT), *n* = 90; aspartate aminotransferase (AST), *n* = 90; alkaline phosphatase (ALP), *n* = 90; gamma glutamyl-transferase (GGT), *n* = 90; bilirubin, *n* = 90; albumin, *n* = 90; immunoglobulin M (IgM), *n* = 90; immunoglobulin G (IgG), *n* = 90; antinuclear antibodies (ANA), *n* = 84; anti-Ro/(Sjögren’s syndrome) SSA antibodies, *n* = 17; anti-SSA 52-60, *n* = 14; anti-La/SSB antibodies, *n* = 14; anti-topoisomerase (Scl) 70 antibodies, *n* = 16; anticentromere antibodies (ACA), *n* = 10; anti-glycoprotein (gp) 210 antibodies, *n* = 15; anti-Sp100 antibodies, *n* = 14; anti-circulating anticentromere (CENP)-A antibodies, *n* = 8; anti-CENP-B antibodies, *n* = 12; anti-double stranded deoxyribonucleic acid (dsDNA) antibodies, *n* = 27; anti-U1-(ribonucleotide protein) RNP antibodies, *n* = 9; anti-Sm-ribonucleoprotein (RNP) antibodies, *n* = 15; anti-promyelocytic (PML) antibodies, *n* = 14; antineutrophil cytoplasmatic antibodies (ANCA), *n* = 20; perinuclear antineutrophil cytoplasmatic antibodies (pANCA), *n* = 20; anti-Myeloperoxidase (MPO) antibodies; *n* = 13; anti-(proteinase) PR 3 antibodies, *n* = 11; antimitochondrial (AMA) antibodies, *n* = 67; AMA-M2 antibodies, *n* = 36; AMA-M2 3E antibodies, *n* = 20; anti-(smooth muscle actin) SMA antibodies, *n* = 60; anti-liver kidney microsomal (LKM) 1 antibodies, *n* = 55; anti-liver cytosol 1 (LC1), *n* = 17; anti-soluble liver antigen/liver-pancreas (SLA)/LP antibodies, *n* = 62; anti-histidyl transfer RNA synthetase (Jo-1) antibodies, *n* = 17.

#### Appendix A.1.2. Methods Applied for Testing on Autoantibodies

ANA: Indirect Immunofluorescence (IIF) on HEp-2 cells. Results are typically reported as a pattern (e.g., homogeneous, speckled, nucleolar) and positive dilution threshold is ≥1:80.

AMA: IIF on rodent kidney, stomach, and liver tissues. This method is used to detect a characteristic mitochondrial pattern. Positive or equivocal findings are confirmed with Enzyme-linked Immunosorbent Assay (ELISA) for specific antibody determination (AMA-M2) or Immunoblot (AMA-M2 and AMA M2-3E). Positive dilution threshold is ≥1:80.

Anti-Ro/SSA; SSA 52-60; Anti-La/SSB; Anti-Scl 70; Anti-CENP-A; Anti-CENP-B; U1-RNP; Sm-RNP; Anti-MPO; Anti-PR 3; Anti-SLA/LP; Anti-Jo1; AMA; AMA-M2; pANCA: ELISA.

ACA: IIF on HEp-2 cells showing a characteristic centromeric pattern. Confirmation is made with ELISA. Positive dilution threshold is ≥1:80.

Anti-dsDNA: Crithidia luciliae Immunofluorescence Test (CLIFT) or ELISA.

p-ANCA: IIF on neutrophil granulocytes for initial diagnosis (showing, for example, cytoplasmic or perinuclear patterns). Positive or equivocal findings are confirmed with ELISA for specific antibody determination (MPO or PR3). Positive dilution threshold is ≥1:80.

Ro-52; gp210; Sp100; PML; AMA-M2; AMA M2-3E: Immunoblotting (Western Blot).
